# Effect of Nb addition on the size and morphology of the β-Fe precipitates in recycled Al-Si alloys

**DOI:** 10.1038/s41598-021-89050-5

**Published:** 2021-05-05

**Authors:** Carlos Jr. Narducci, Gabriela Lujan Brollo, Rafael Humberto Mota de Siqueira, André Silva Antunes, Antonio Jorge Abdalla

**Affiliations:** 1 Aeronautics Institute of Technology – ITA, São José dos Campos, SP Brazil; 2 Faculty of Mechanical Engineering, University of Campinas – UNICAMP, Campinas, SP Brazil; 3 Department of Photonics, Institute for Advanced Studies – IEAv, São José dos Campos, SP Brazil; 4 Federal Institute of São Paulo – IFSP, Itaquaquecetuba, SP Brazil; 5 1249, Rua Alto Garças, Cidade Patriarca, São Paulo, SP Brazil

**Keywords:** Engineering, Materials science

## Abstract

The recycled Al alloys have a Fe level above the recommended limits, leading to the precipitation of β-Fe intermetallic particles in their microstructure. The brittle β-Fe particles show a rough morphology in the form of highly-faceted platelets, which is detrimental to the alloy's mechanical performance containing these precipitates. This work analyses the possible interactions of the addition of 0–1 wt% of the grain refiner Nb + B to the morphology and size of β-Fe precipitates in alloys melted with Al-(7, 9, 12) wt% Si and 1 wt% Fe. The results indicate that the addition of the Nb + B inoculant revealed a significant change in the precipitates' size and morphology, which have become remarkably refined and spheroidized. Moreover, deepening the study through qualitative and quantitative analyses, it was found that the behaviour of the β-Fe precipitates follows an exponential decay with the increasing addition of Nb + B, a curve analogous to the primary α-Al grain refinement one, revealing a direct correlation between the events. Finally, it was possible to suggest a mechanism that shows how the phenomenon of morphological transformation of the β-Fe precipitates occurs in the material with the addition of the Nb + B inoculant.

## Introduction

In the aluminium casting business, the use of secondary Al (obtained from recycling) represents a competitive advantage due to the lower cost when compared to the production of alloys using primary Al. Green (2007) reports that the production of aluminium from bauxite ore attracts an energy consumption of about 186 MJ/kg of metallic aluminium; however, this consumption could be reduced to 10–20 MJ/kg by recycling the discarded aluminium products, saving 95% of the energy used as compared to making the metal from the original bauxite ore^1^. However, the secondary Al has Fe contents levels above the recommended limit, this restricts its use in structural and safety applications due to the brittleness induced by precipitates rich in Fe originated in alloys' microstructure contaminated with Fe. It would be necessary to quantify the cost of removing Fe in the Al alloy to compare the advantage of using recycled Al. Taylor (2012) demonstrates that in Al-Si alloys with 5 wt% Si, the critical iron concentration is ~ 0.35%; at 7% Si, this content rises to ~ 0.5% of Fe; at 9% Si the acceptable Fe content is ~ 0.6%; and at 11% Si it can reach ~ 0.75% of Fe. These levels are calculated through the section of the liquidus projection of the Al-rich corner of the Al-Si-Fe ternary phase diagram that highlights the existence of a critical iron content (Fe-critical), in which the critical iron level is directly related to the silicon concentration of the alloy^2^. However, it should be noted that the Fe-critical level may be interesting when the casting process is by way of high pressure (HPDC). It will be necessary to correlate Fe's level in the material with the desired application. Mahta (2007) reports that the presence of Fe, even in small amounts, degrades the mechanical properties of aluminium alloys such as tensile strength and fatigue, tenacity to fracture, and especially the elasticity, due to the formation of the β-Al_5_FeSi (β-Fe) intermetallic phase, with a plate-like or needle-like morphology, observed on the surface of the broken material in the tensile test. Mahta proposed as a modification mechanism the addition of some elements, with Mn being the most used for alloys with Fe-critical, due to the formation of α-Al_15_ (FeMn) _3_Si_2_, i.e., replacing β-Fe with α-Fe^3^. However, with this mechanism a new problem may arise due to the so-called sludge formation^4^, which ends up damaging the mechanical properties of the material. Representative mechanical tests would be necessary to evaluate the precise conditions under which Mn's use can minimize the effect of β-Fe precipitates on the material structure.

The most straightforward approach to counter the level of iron impurity is to dilute the recycled aluminium using primary aluminium, but eventually, it makes the Al-Si alloy more expensive. Basak (2016) suggested the fragmentation theory as the main mechanism for refining the primary β phase, based on experimental evidence^5^. However, in this theory, morphological change of β-phase can be induced by suitable heat-treatment, however, it is only recommended in the low-Si high-Fe content recyclable Al-Si alloy, in addition to increasing the cost of the final product. Basak also proposed another approach, the gravitational segregation, but this is not a good productivity process. A cost–benefit analysis would be necessary to show the advantages of using these techniques economically. With this, there is a real need to look for a new alternative to deal with the high iron content in recycled aluminium alloys.

From the classical theory of heterogeneous nucleation used in the phenomenon of grain refinement in Al-Si alloys, the possibility that one of the effects of this transformation could be the most homogeneous distribution of solute elements arise, in the case Fe, in the solidification fronts during the eutectic phase of the material transformation, thus avoiding the interconnectivity of the Fe elements dispersed in the liquid metal in the shape of a needle or thick platelet. Nowak (2014) reports that niobium-based compounds, through the mechanism of heterogeneous nucleation of grains, are highly effective in refining the α-Al dendrites grains in Al-Si alloys with Si contents greater than 6 wt%, showing through experiments with the addition of the Nb + B-based inoculant, the grain size less dependent on the solidification process (i.e. cooling rate), this leads to not only the finer primary α-Al grain but also to a finer Al-Si eutectic phase due to the more homogeneous distribution of the alloying elements in the solidification front and eutectic pools, resulting in significantly refined microstructural features (grain size and eutectic phase or secondary phases)^6^. However, the study lacks depth or novelty to characterize the real change in intermetallic precipitates' morphology (β-Fe).

Despite extensive research and consolidated knowledge about the use of Nb + B as a grain refiner, there are no studies in the literature that directly associate the effect of adding Nb to the morphology of β-Fe precipitates, measuring these effects in qualitative and quantitative terms and correlating the size of the primary α-Al grain with the size of the β-Fe precipitates and trying to understand the reasons that lead to its self-flagellation. Hence, this is the objective of the present study.

## Experimental procedure

The Al-(7, 9, 12)wt%Si-1wt%Fe alloys were used as base alloys to analyse the effect of Nb addition on the morphology of the β-Fe precipitates. Table 1 is presented the level of Nb + B added to each alloy.

**Table 1:** Nb-B level range added to the Al–Si–Fe alloys.

Combination	I	II	III	IV
Nb (wt.%)	0	0.02	0.10	1.00
B (wt.%)	0	0.0025	0.0125	0.125

The studied alloys were cast in an open resistive oven with a tilting ceramic crucible with a melting capacity of 3 kg Al. The primary Al (supplied by HYDRO), was heated and stabilized at 850 °C (an immersion pyrometer controlled the temperature). Then, were added to the molten metal the Si alloying elements (supplied by LIASA) and Fe (supplied by MEXTRAMETAL). After addition each element, a 1-hour hold was applied to ensure complete dissolution. After that, the bath was homogenized by mechanical (manual) stirring for 30 s. Degasification was then applied by adding hexachloroethane tablets (supplied by ALFA TREND), to the bath in a proportion of 0.1 wt%, followed by manual slag removal. The verification of the base alloy was done by atomic absorption spectrometry. Can be seen in Table 2 results.

**Table 2:**
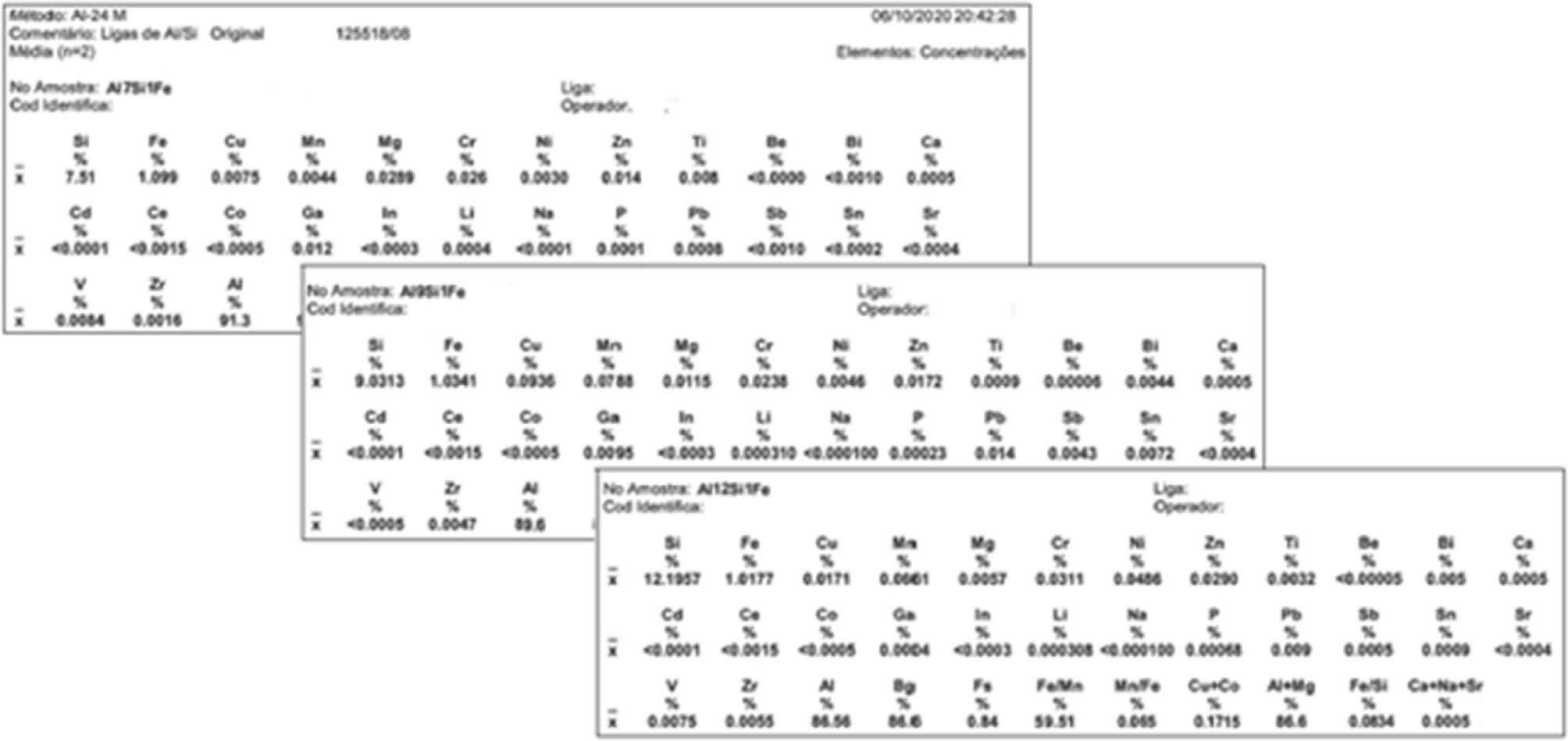
Atomic absorption spectrometry report (analysed by LATASA).

After the preparation of each Al-Si-Fe based alloy, progressive amounts of Nb were added to the bath in the form of the Al-4Nb-0.5B master alloy (provided by the Brazilian Metallurgy and Mining Company—CBMM). For each Nb level, homogenization was achieved by 30 s of manual stirring and a new stabilization at 720 °C, followed by a second homogenization and sample collection.

**Figure 1:**
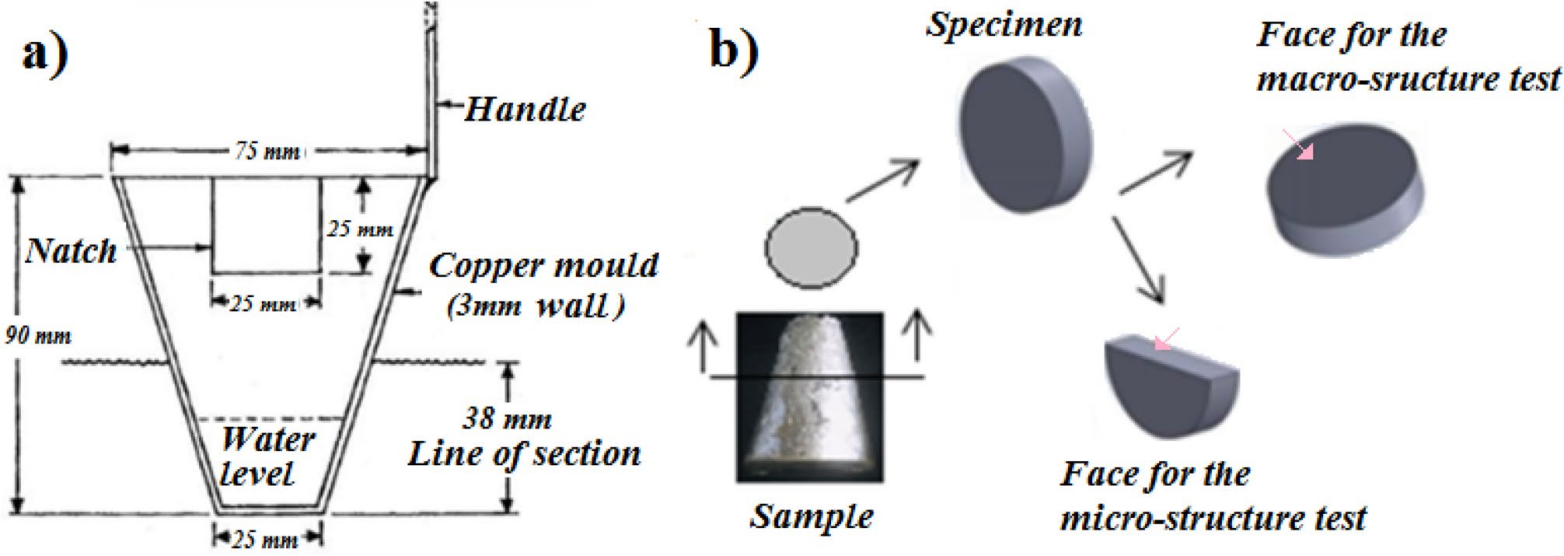
Illustrative sketch. (**a**) Mould ladle. (**b**) Cut of the sample.

Seven samples were obtained from each batch. The extraction of the samples was performed according to the procedure described by the standard mould technique, Test Procedure—1 (TP-1)^7^, as follows:

The TP-1 mould ladle (Fig. 1a) was placed inside a furnace for heating at 350 °C.

The TP-1 mould quench tank's water flow rate was set to 3.8 l min. This flow rate resulted in a 25 mm submersion from the bottom ladle.

The test sample removed from the TP-1 conical mould was cut according to the standard procedure (38 mm above the base) as illustrated in Fig. 1. The cooling rate at this position corresponds to 3.5 °C/s.

Various factors that affect the solidification microstructure of a given alloy, but one of the most important is the cooling rate. By keeping the cooling rate constant, we can examine the effect of heterogeneous additions on the grain size. The standard Test Procedure-1 (TP-1) mould technique was used to maintain a constant cooling rate.

The cross-sectional surfaces were prepared with 2400 mesh sandpaper, without polishing, to check the material's macrostructure on one side of the sample. After metallurgical preparation, the samples were attacked for 15 seconds with a Poulton acid solution (60% HCL at 37%; 30% HNO_3_ at 65%; 5% HF at 50% and 5% H_2_O), then washed in water for 20 seconds. Cleared then with (67% HNO_3_ at 65%; 5% HF at 50% and 13% H_2_O) and 15 seconds. On the other side of the samples, the surfaces of the cross section were prepared with 2400 mesh, polished with 1 μm alumina suspension, and then chemically etched with the Keller reagent (95.0 ml of distilled water, 2.5 ml of HNO_3_, 1.5 ml of HCl and 1.0 ml HF) to reveal their microstructural constituents.

The macrostructure (grain size) was examined in an optical microscope with polarized light and a plate with filter and differential interference contrast (DIC) to reveal the limits of the primary grains of α-Al. The average grain size (G) measurement was carried out using the linear intercept method according to the standard ASTM E112-10, 1996^8^ and calculated the measurement by counting the number of grains that intersect a known size line. The alloys' microstructure (β-Fe intermetallic precipitates) was analysed by X-ray Dispersive Energy Spectroscopy (EDS). The analysis was performed on the samples with a Tescan scanning electron microscope with an EDS detector analyser from Oxford Instruments, to obtain the Al, Si and Fe spatial spectrums. From random positions of the samples were the images taken.

The microstructural evolution of the β-Fe particles was analysed using the Image J Fiji 1.46 user guide (2017)^9^, using the Fe spatial spectrum of the SEM–EDS images. The following parameters were measured: size (in μm^2^), represented by the area occupied by each particle in the microscopic image; the maximum Feret diameter (in μm), which is the largest distance between two points in the particle outline, also known as the maximum caliper diameter; the fraction of the particles in the alloys (% area), estimated as the ratio between the area occupied by these particles and the total area of the respective microscopy image; and the shape factor, circularity (without dimension), represented by the equation C = (4πA)/P^2^, where C is the circularity, A is the area and P the perimeter of the particle (C = 1: sphere; C = 0: needle). Particles smaller than 2 μm were not considered in the calculations to avoid including background noise in the analysis.

## Results and discussion

**Figure 2:**
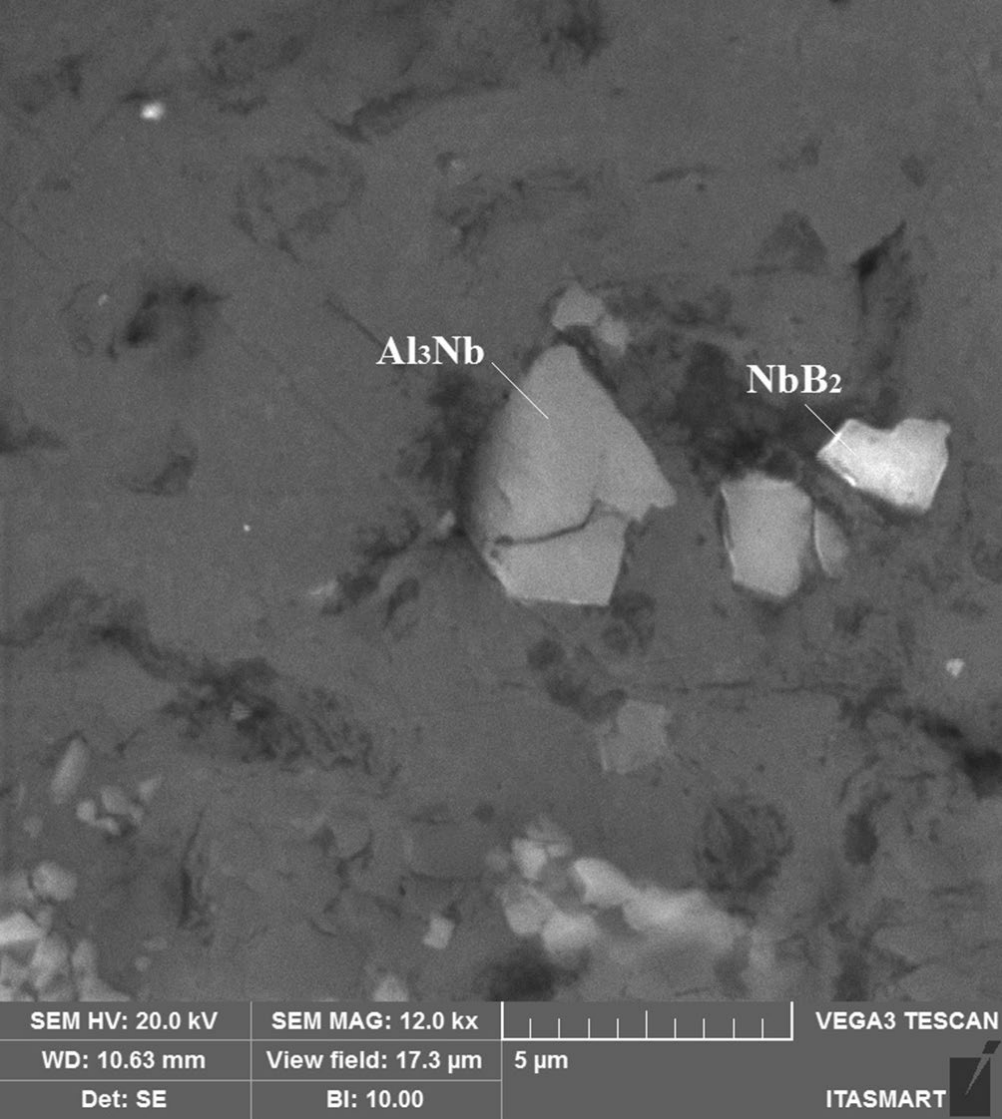
Al_3_Nb and NbB_2_ substrates identified in the scanning electron microscope (SEM), with a detector analyser (SEM–EDS), present in the core of the primary α-Al grain, found in the Al7Si1Fe alloy, with the addition of 0.5% Nb.

The microstructural analysis of the samples identified the substrates responsible for the different nucleation fronts in the material after the addition of the Nb + B inoculant, as shown in Fig. 2.

These substrates are composed of the elements (Al, Nb and B), which react to form niobium aluminides (Al_3_Nb) and niobium borides (NbB_2_). These substrates remain stable after casting the alloy. Based on this, it was possible to verify that they are formed according to Eqs. (1) and (2)^10^:

1 $${\text{Nb }} +_{{3}} {\text{Al }} \to {\text{ Al}}_{{3}} {\text{Nb}}$$

2 $${\text{Al}}_{{3}} {\text{Nb }} + {\text{ AlB}}_{{2}} \to {\text{ NbB}}_{{2}} + {\text{ 4Al}}$$

These Nb-based compounds are the potential nucleation substrates (i.e., inoculants) that promote Al-Si alloys' grain refinement through heterogeneous nucleation. This process is described in the literature as a solid particle, which is still in a liquid phase. When placed in contact with the substrates (Al_3_Nb or NbB_2_), it “wets”, i.e., it spreads and covers the surface, causing the subcooling that is required for grain nucleation^11^. Clusters of AlB_2_ and Al_3_Nb substrates are revealed as crystals that grow along a non-specific direction, without a particular orientation relationship, initiating several nucleation points simultaneously, limiting the size of the α-Al grain dendrites. This phenomenon creates a mechanism that modifying the morphology of 2nd phase precipitates^12^, such as the β-Fe one.

The choice of adding an Al-Nb-B master alloy used in the form of a stick instead of adding Nb powder and KBF_4_, and still with an Nb >> B ratio, comes from the laboratory studies by Bolzoni (2019), who reports that the recovery of B from KBF_4_ was considered to be quite poor. The yield of Nb recovery from the powder was also quite low and variable since Nb oxidizes easily at high temperatures. Moreover, he claims that the use of master alloys with Nb > B performs better than other master alloys with Nb = B or Nb < B, showing that this happens because Nb does not only form borides (NbB_2_), but also niobium aluminides (Al_3_Nb), powerful substrates in heterogeneous nucleation^13^.

**Figure 3:**
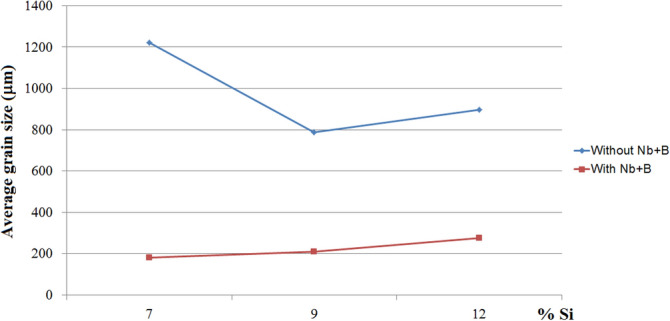
Comparison of the average variation in the primary α-Al grain size with the silicon content, without and with Nb + B. (Microsoft Excel 2010).

The influence of the Si concentration on the material structure was analysed through the macrostructure of the samples, comparing the average grain size without and with the addition of the Nb + B inoculant, revealing that the size of the primary α-Al grain is reduced in a remarkably close order of magnitude in the three spectra of the Si content analysed (i.e., 7, 9 and 12 wt% Si), as can be seen in Fig. 3.

**Figure 4:**
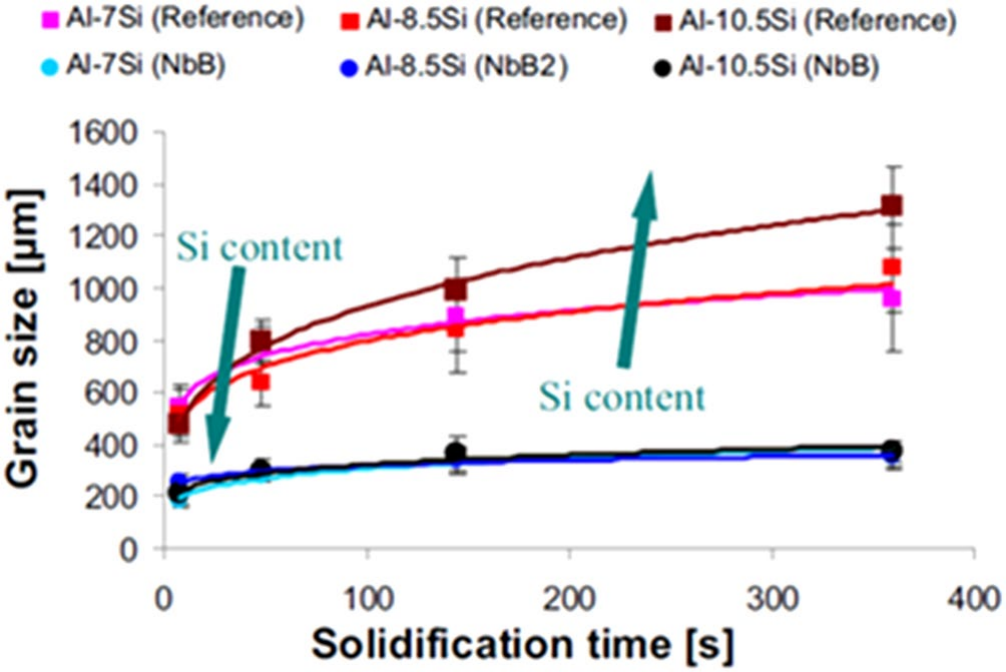
Variation in grain size versus solidification time of Al-Si alloys without (reference) and with inoculation (Nb + B). Adapted from Ref.^10^.

The study by Bolzoni and Babu (2016) also confirms this result, in which the authors thermally analysed Al-Si alloys without inoculation and with Nb + B inoculation, showing the influence of the Si concentration on the addition of heterogeneous nuclei projected in the solidification phenomena (i.e., grain nucleation of the primary α-Al grains and nucleation of the secondary eutectic phase of Si), as shown in Fig. 4^10^.

**Figure 5:**
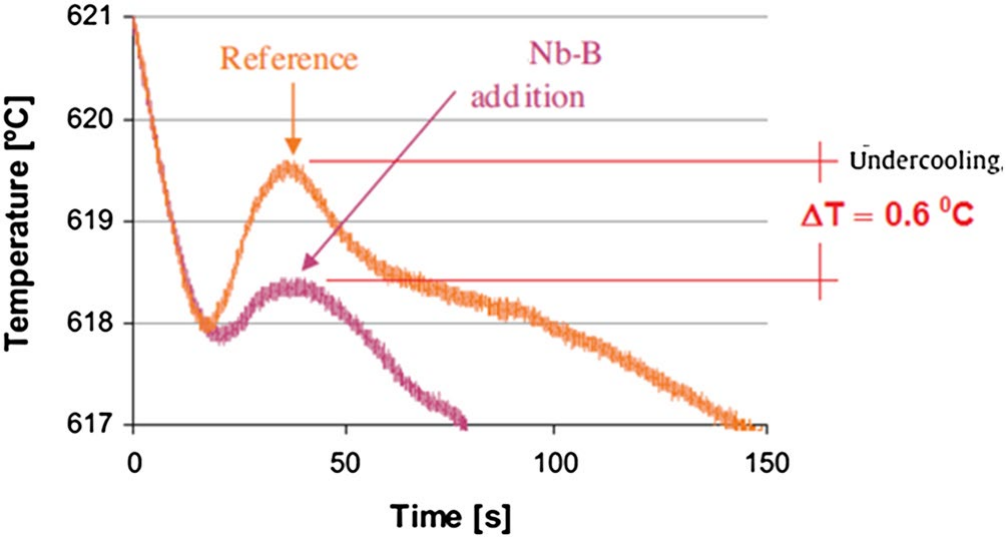
Comparison of the cooling curve of the Al-Si alloy, with and without Nb + B. Adapted from Ref.^14^.

For reference alloys, the grain size is significantly affected by the solidification time and the Si content. After inoculation, the grain size is much less dependent on the solidification conditions and there is no significant dependence on the Si content. The cooling curves elements are moved to a lower solidification temperature, according to the binary diagram of the Al-Si alloys^10^. The effects of Nb on the transformation of the primary α-Al grain and eutectic phases were discussed in the study by Bolzoni and Babu (2015), in which they show the comparison of the cooling curves of the Al-Si alloy without and with the addition of the Nb + B inoculant, as shown in Fig. 5.

**Figure 6:**
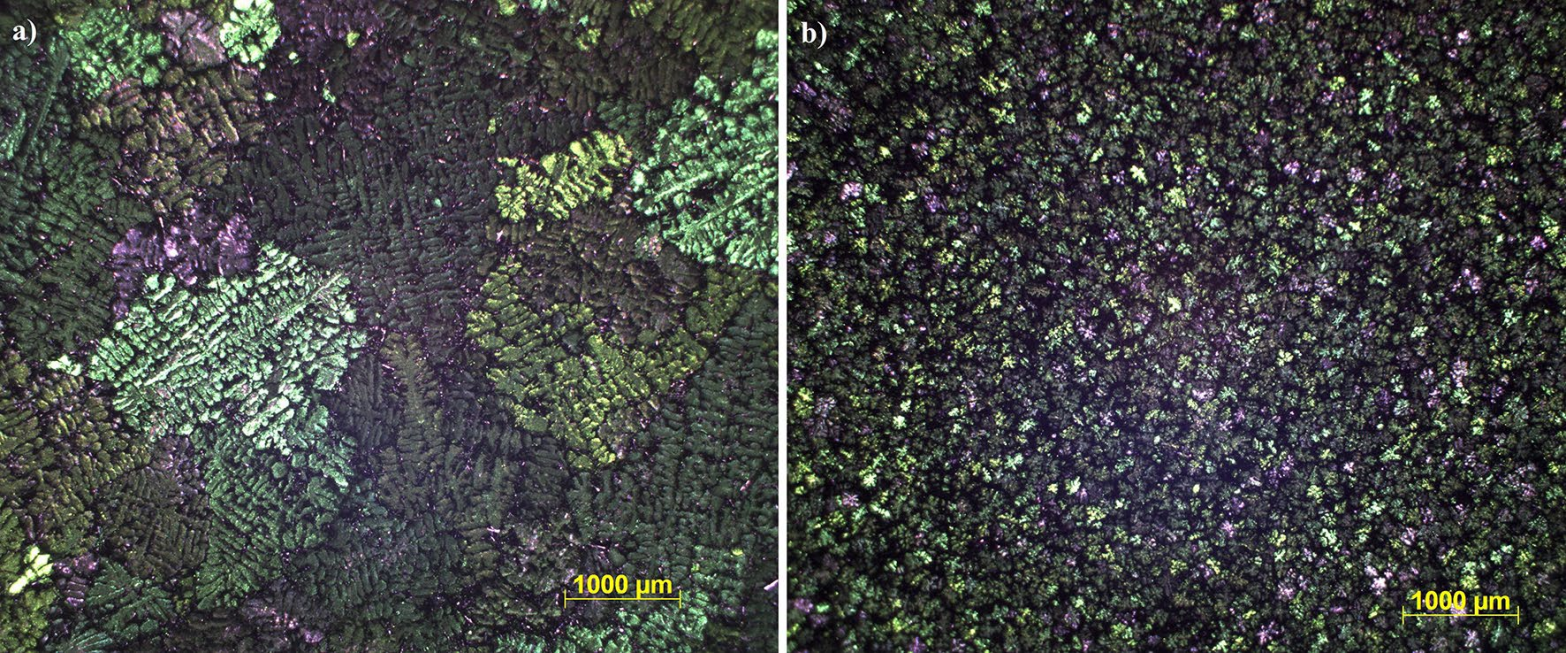
Primary α-Al grain. (**a**) Without the addition and (**b**) with the addition of the Nb + B inoculant (0.05 wt% Nb).

The analyses show that there is a thermodynamic barrier of nucleation in the alloy without the addition of Nb + B due to the slow cooling employed during the thermal analysis; the primary aluminium grains start to grow, then the temperature rises reaching a peak known as recalescence. Based on the comparison of the material's cooling curve without and with the addition of Nb + B, where it is shown that the shape of the curves is not changed, and the minimum temperatures are comparable, but the recalescence temperature is changed. Consequently, the undercooling after the adding of the Nb + B inoculant is lower. The reduction of the undercooling generated during the solidification of the material determines the condition of the primary α-Al grains nucleation, happening simultaneously in several points in the material^14^, resulting as a direct effect in the reduction in grain size of the primary α-Al grain, as shown in Fig. 6.

The study results showed that the physical mechanisms that influenced the morphology of the material’s microstructure are compatible with the current literature's results. However, it is necessary to deepen the study, seeking to know qualitatively and quantitatively the β-Fe precipitates' morphology to understand their formation. That is the purpose of this study.

**Figure 7:**
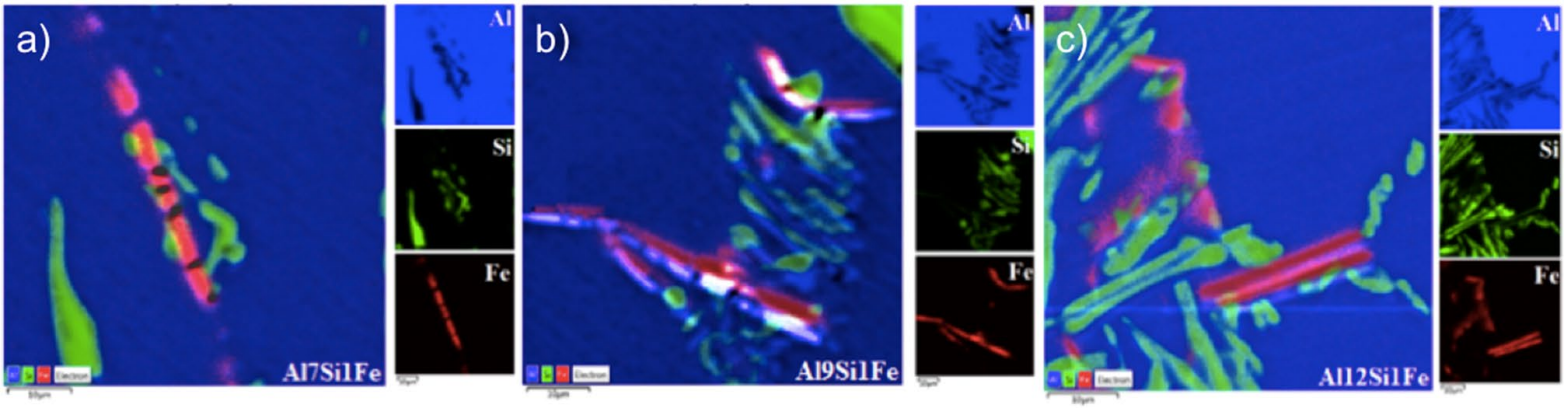
SEM–EDS maps highlighting typical eutectic precipitates present in the alloys containing 7 (**a**), 9 (**b**) and 12 wt% Si (**c**) and 1 wt% Fe, without Nb addition. Al, Si and Fe are highlighted in blue, green and red, respectively.

In this sense, the presence of β-Fe precipitates in the samples produced without the addition of Nb + B (Fig. 7), obtained by SEM–EDS maps, was analysed.

The images in Fig. 7 show that the β-Fe precipitates in their primitive formation (a morphology typical of thick platelets), are present in the three alloys studied and are compatible with the images shown in the study by Mahta et al. (2007), in which he states that this phase has long been thought to be brittle and responsible for the inferior mechanical properties (in particular, elasticity) of the aluminium cast alloy^3^.

**Figure 8:**
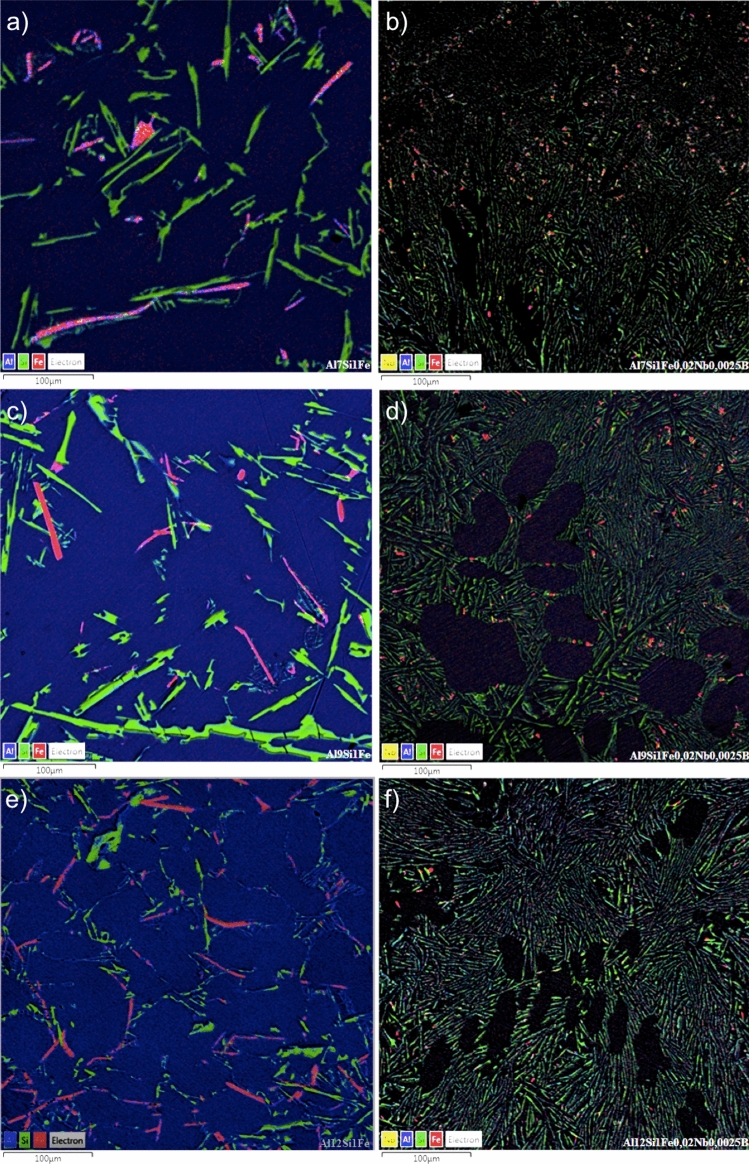
SEM–EDS images displaying the microstructure of the alloys containing 7 (**a**, and **b**), 9 (**c**, and **d**) and 12 wt% Si (**e**, and **f**) and 1 wt% Fe, without Nb addition (**a**, **c**, and **e**) and with 0.02 wt% Nb (**b**,** d**, and **f**). Al, Si and Fe are highlighted in blue, green and red, respectively.

Figure 8 presents SEM–EDS images showing the casting samples' microstructure without (a, c, and e) and with Nb + B (b, d, and f). The colour of the Al element has been suppressed in some images for better visualization of the eutectic phase.

Regardless of the Si content, the microstructures of the alloys are similar. In the alloys without Nb addition (above in Fig. 8a, c, and e), β-Fe particles are present in the form of thicker needles and platelets. A slight addition of 0.02 wt% Nb and 0.0025 wt% of B was enough to drastically change the morphology and size of the β-Fe precipitates, which became very refined and spheroidized (below in Fig. 8b, d, and f).

**Figure 9:**
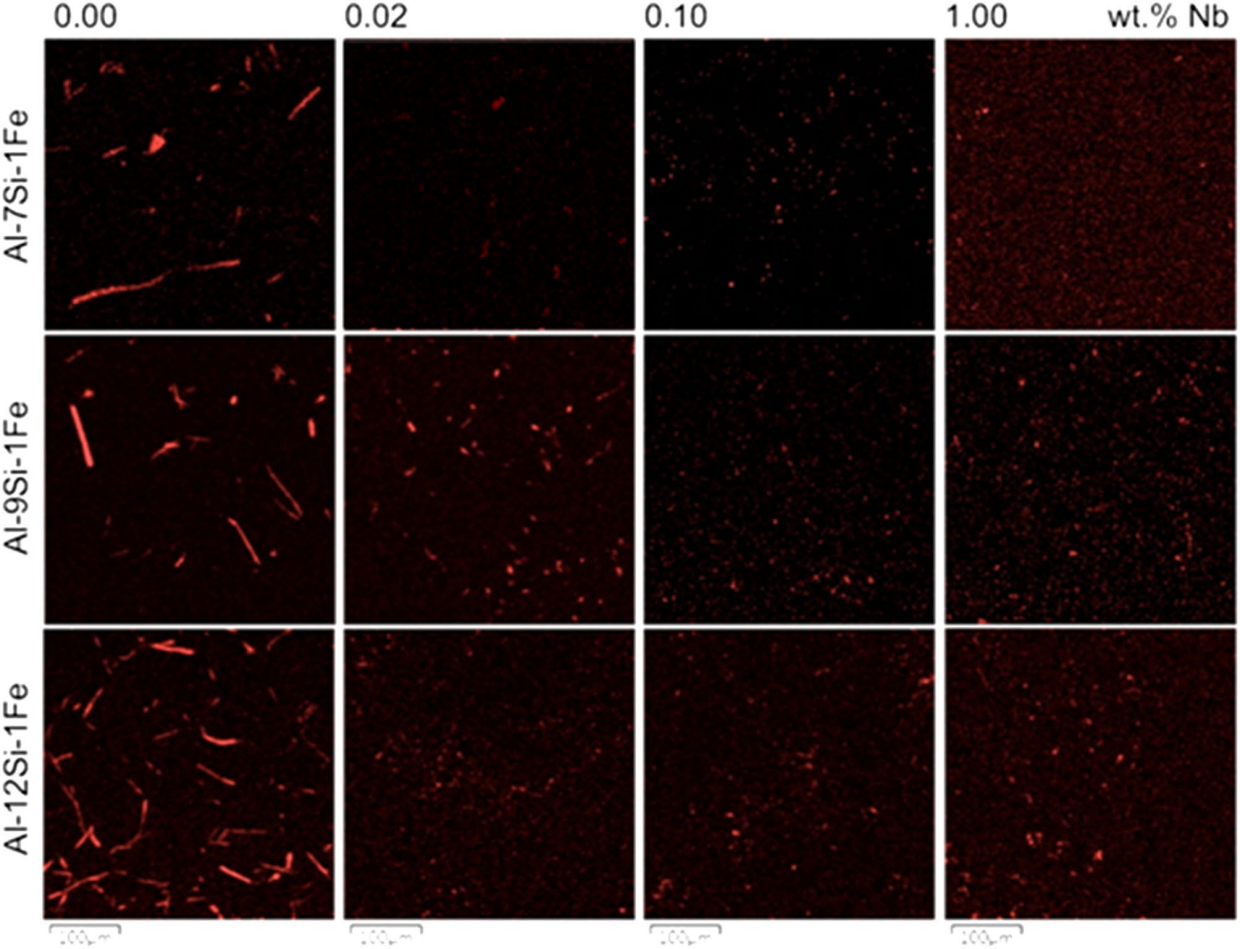
Fe-rich spectrum of the SEM–EDS images for the alloys containing 7, 9 and 12 wt% Si and 1 wt% Fe, without Nb addition (left) and with 0.02–1.00 wt% Nb and B as per stoichiometric calculation.

To evaluate the effect of the Nb + B inoculant in a wide range, from 0.02 to 1.00 wt% Nb and B as per stoichiometric calculation, Fig. 9 shows the Fe-rich spectrum of SEM–EDS images for the studied alloys, presenting the total range of the Nb + B content analysed.

Based on the qualitative results found in the samples, statistical analyses were performed to quantify better and characterize the change in the size and morphology of the β-Fe precipitates, as can be seen in Table 3.

**Table 3:** Microstructural parameters (average ± standard deviation) of β-Fe precipitates for all the studied alloys and Nb contents.

Alloy	Nb (wt%)	n*	Size (µm^2^)	Feret (µm)	Area (%)	Circularity (–)
AI7Si1Fe	0.00	93	33.7 ± 102.0	8.2 ± 15.8	1.86	0.82 ± 0.24
	0.02	71	11.9 ± 13.1	5 1 ± 2 3	0.50	0.85 ± 0.17
	0.10	85	5.8 ± 4.6	3.7 ± 1.5	0.29	0.92 ± 0.13
	1.00	35	6.6 ± 7.0	3.3 ± 2.1	0.14	0.93 ± 0.14
AI9Si1Fe	0.00	41	72.2 ± 138.0	15.6 ± 20.9	1.76	0.69 ± 0.27
	0.02	73	25.2 ± 23.0	7.9 ± 4.3	1.09	0.79 ± 0.18
	0.10	41	5.8 ± 5.4	3.6 ± 2.0	0.14	0.93 ± 0.13
	1.00	58	6.5 ± 5.8	3.8 ± 1.9	0.23	0.92 ± 0.14
AI12Si1Fe	0.00	124	49.9 ± 88.5	12.8 ± 14 3	3.68	0.67 ± 0.29
	0.02	57	8.3 ± 7.5	4.4 ± 2.1	0.28	0.88 ± 0.15
	0.10	74	10 0 ± 12.8	4.6 ± 3.0	0.44	0.87 ± 0.18
	1.00	38	9.3 ± 7.3	5.2 ± 2.3	0.26	0.83 ± 0.17

*Sample size (number of particles) analyzed.

**Figure 10:**
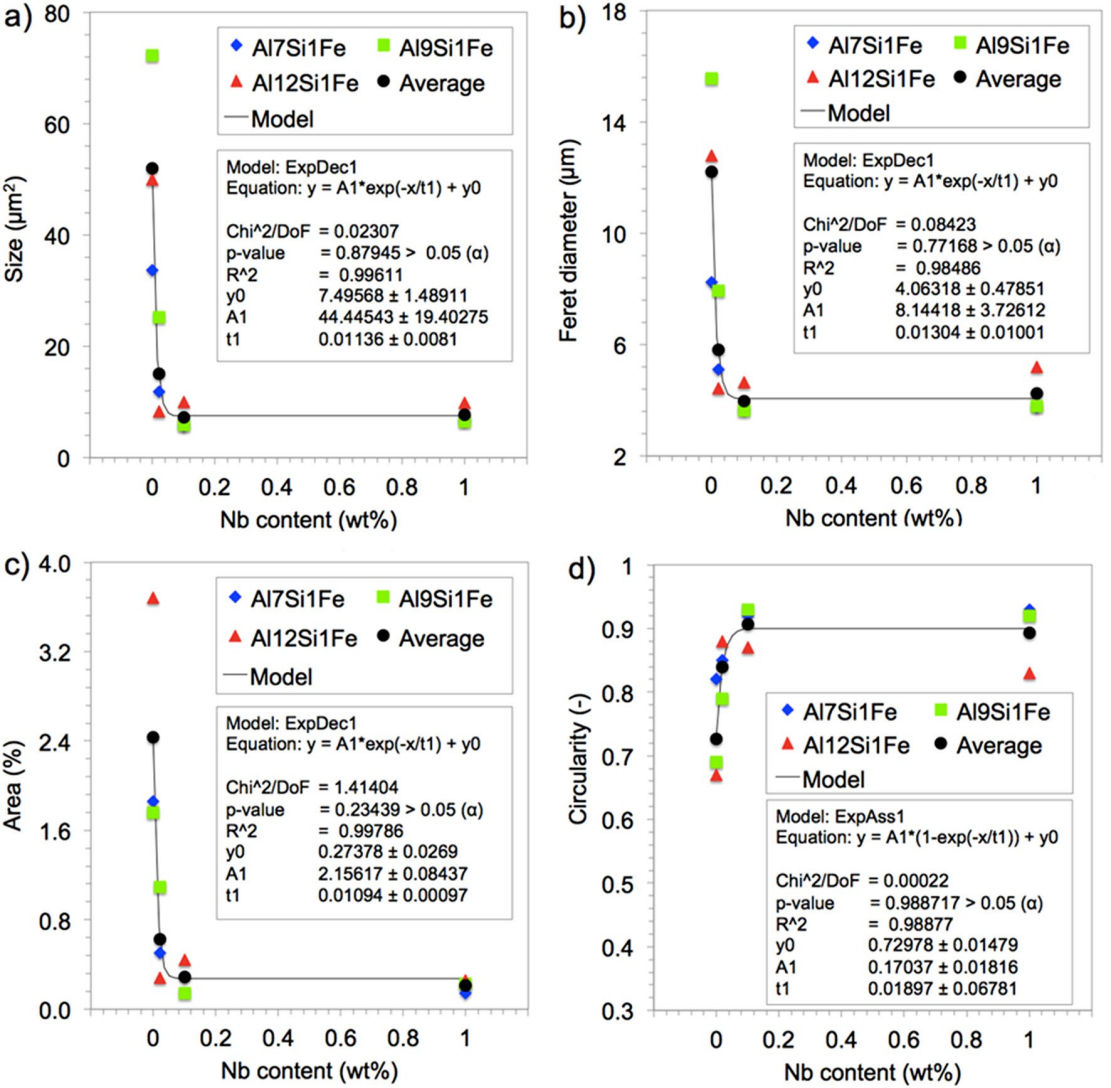
Microstructural parameters of β-Fe particles for all alloys and Nb contents analysed. Experimental data for size (**a**), Feret's diameter (**b**), % area (**c**) and circularity (**d**) are shown together with the respective modeled curve. (Image J Fiji 1.46 user guide—2017).

Table 3 shows the microstructural parameters (average ± standard deviation) of β-Fe precipitates for all studied alloys and the Nb contents, measured using the images in Fig. 9. The data of Table 3 were plotted in the graphs of Fig. 10 for better viewing: size (a), max. Feret’s diameter (b), % area (c) and circularity (d). Together with the experimental points, the curve corresponding to the mathematical models adjusted to the data is also shown in Fig. 10 (black curve). The model equation parameters and two quality criteria of the adjustment between the experimental data and model (R^2^ and Chi^2^) are also presented for each graph.

The equations demonstrate that, regardless of the Si content, the microstructural parameters of the β-Fe precipitates showed similar behaviour with the increase in Nb content, similar to the result presented in the study by Bolzoni and Babu (2016), for grain refinement in Al-Si alloys, as previously commented^12^. The particle size (Fig. 10a), Feret's diameter (Fig. 10b) and % area (Fig. 10c) obey a first-order exponential decay represented by Eq. (3) below. The circularity (Fig. 10d) resulted in a first-order exponential association represented by Eq. (4), also shown below.

3 $${\text{y}} = {\text{A}}_{1} *{\text{e}}_{1}^{{( - {\text{x}}/{\text{t}})}} + {\text{ y}}_{0}$$

4 $${\text{y }} = {\text{ A}}_{1} *\left[ {1 - {\text{ e}}_{1}^{{( - {\text{x}}/{\text{t}})}} } \right] + {\text{ y}}_{0}$$

where y is the output, represented by the size in μm^2^, Feret in μm, % area in % (for Eq. 3) and the dimensionless circularity (for Eq. 4), y_0_ is the respective offset for these parameters, A_1_ is the base constant, 1/ t is the rate constant and x is the input, represented by the Nb content in wt%.

The precision of adjustment calculated via both R^2^ and Chi^2^ has showed a suitable statistical correspondence between the experimental data and the theoretical curves, i.e., with R^2^ ≥ 0.98 and p-value (Chi^2^) > α, ensuring acceptance of the null hypothesis.

Physically, the results of the progressive addition of Nb in Eq. (3) showed an exponential decay in the size and morphology of the β-Fe precipitates compared to the alloys without the addition of Nb, varying according to the addition between the studied intervals, as follows: with the addition of Nb between 0 to 0.02%, a reduction on an average of 3.56 times in size, 2.22 times in Feret and 4 times in area %; with the addition of Nb between 0.02 to 0.1%, a reduction on an average of 9.81 times in size, 3.13 times in Feret and 8.28 times in area %; however, with the addition of Nb between 0.1 to 1%, the parameters remained practically the same as the previous level, probably due to the temperature used during the melting of the material which only allowed the maximum solubility of 0.1 wt% Nb in Al. Thus, the formation of substrates and the appearance of new cores is limited to the additions of more than 0.1 wt% Nb. Nb solubility values in Al are known and available in the literature^6^.

Physically, the results of the progressive addition of Nb in Eq. (4) showed an opposite behaviour in circularity, indicating an expressive spheroidization of the particles with the increase of the Nb content, an exponential association model. Also varying according to the addition between the studied intervals, notably: with the addition of Nb between 0 to 0.02%, an increase on an average of 1.18 times in circularity; with the addition of Nb between 0.02 to 0.1%, an average of around 1.26 times in circularity; with the addition of Nb between 0.1 to 1%, the circularity remained practically the same as the previous level, with the same explanation given for the other parts of Eq. (3).

A remark should be made concerning the exponential decay observed in the % area occupied by the β-Fe precipitate when increased the Nb content (Fig. 10c). This trend may be caused because β-Fe particles are reduced in size to a level with progressive Nb additions that the precipitates could not be visualized in the magnification allowed by the SEM–EDS technique used here. If this is the case, this effect leads to the erroneous impression that the Nb addition causes a reduction in the amount of β-Fe particles formed in the sample. Or the solute elements were dispersed in the matrix of Al, reducing the formation of precipitates, which suggests that should be completed the SEM–EDS technique with a more detailed investigation on this subject via TEM analysis, which is outside the scope of this study.

Nb's addition somehow changed the precipitation dynamics of the β-Fe particles, leading to a smaller size of particles formed with spheroidized morphology, it is probably on account of a more homogeneous distribution of the solute elements (Fe or Si), rejected in the solidification front and eutectic pools, but now trapped among the diverse and reduced dendritic arms of α-Al grains, thus avoiding approximations, clusters, or interconnectivity between the solute elements.

**Figure 11:**
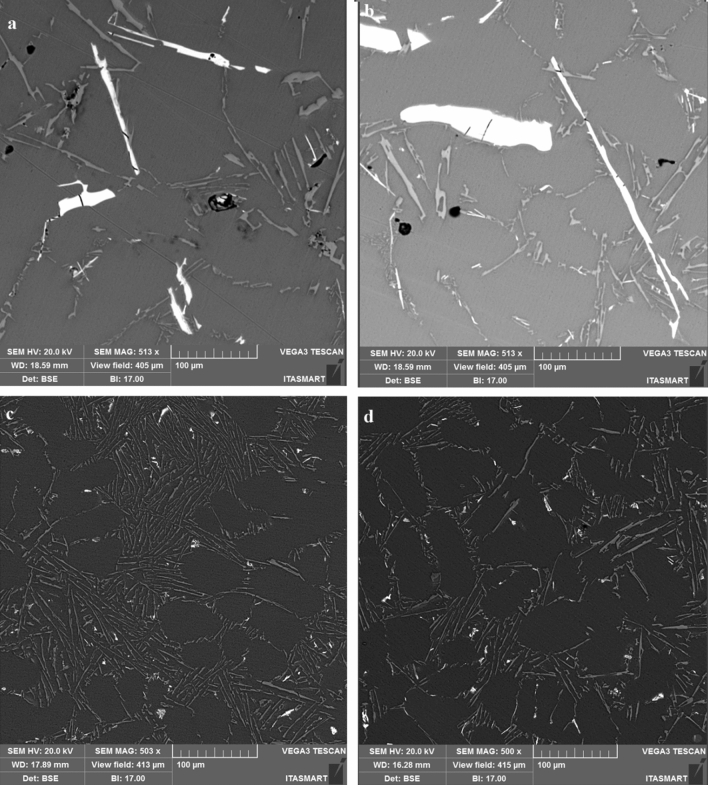
Image showing the morphology of the β-Fe (light grey) spectra before the addition of Nb + B (**a**, and **b**) and after the addition of 0.1%Nb + 0.0125%B (**c**, and **d**).

Figure 11 is another confirmation of these effects, through the β-Fe spectra highlighted by the light grey colour, in the samples without the elongated Nb + B inoculant, Fig. 11(a, and b), and with the addition of the Nb + B inoculant of reduced size and spheroidized shape, Fig. 11(c, and d).

**Figure 12:**
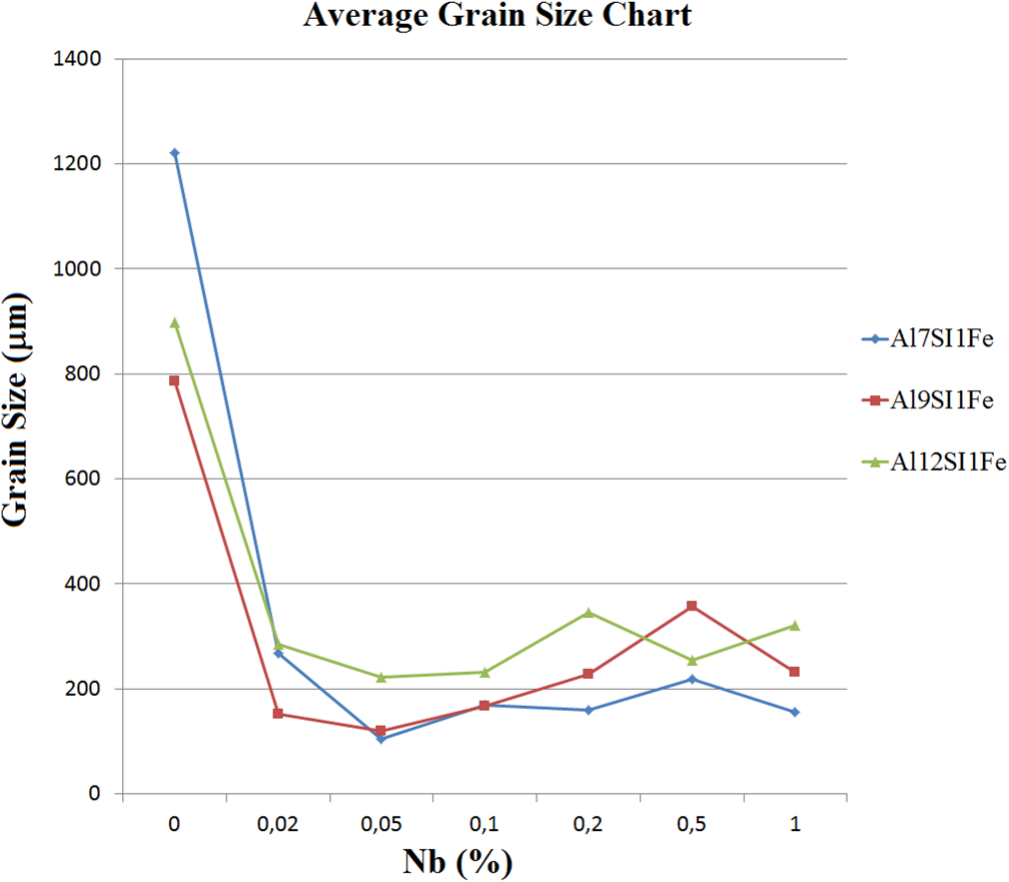
Average Grain Size according to Nb wt% (Microsoft Excel 2010).

For the analysis of the relationship between β-Fe precipitates' behaviour and their relationship with the refinement of the primary α-Al grain, as a function of the level of Nb added to the material, the average grain size was calculated, as shown in Fig. 12.

**Figure 13:**
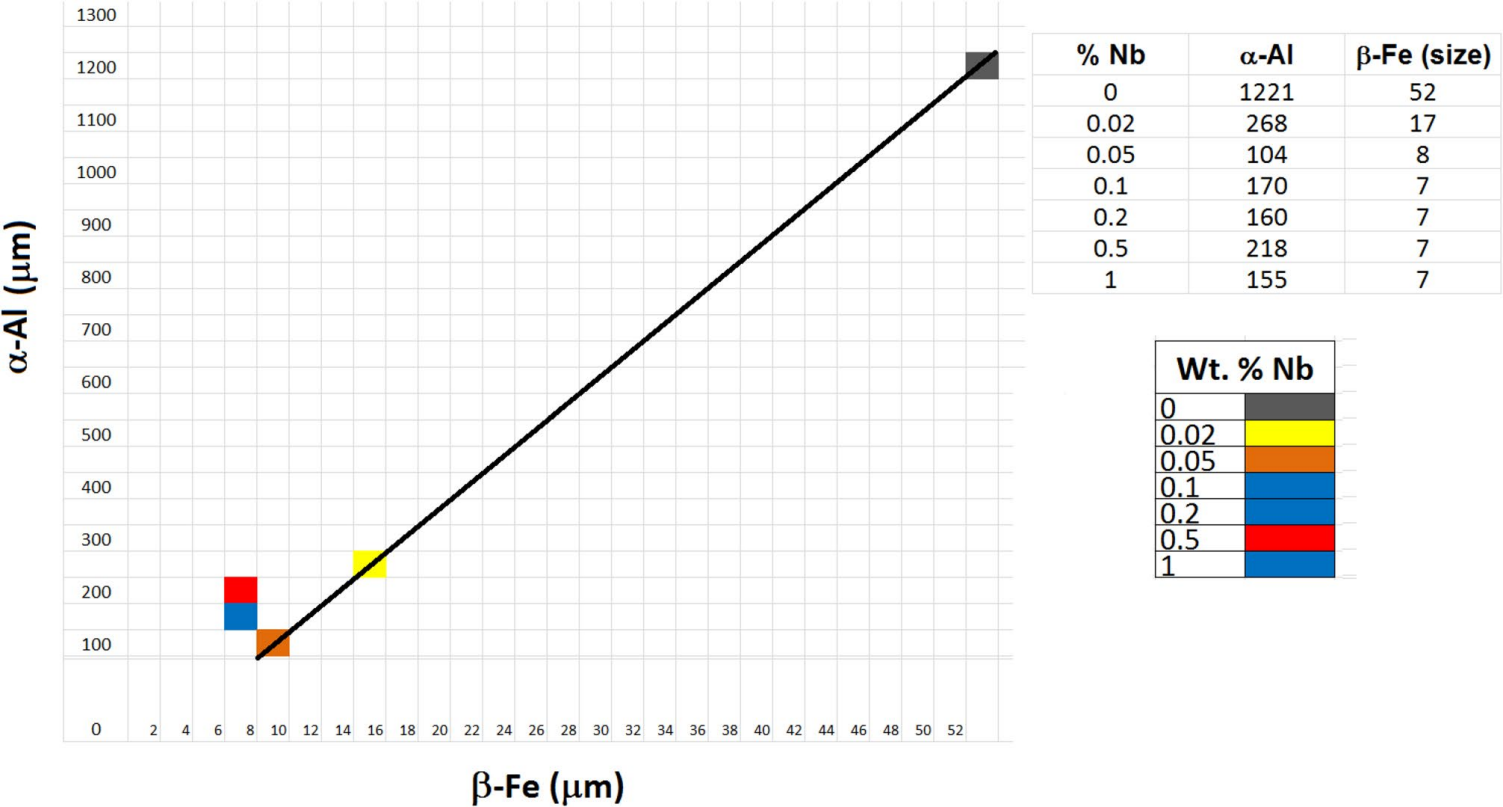
Average size of β-Fe precipitates versus the average size of primary α-Al grains (Microsoft Excel 2010).

It can be observed that the graph of the average grain size is similar to the graphs of the microstructural parameters of the β-Fe particles, following an exponential decay in the α-Al grain size after the addition of the Nb + B inoculant. The study between the curves reveals a direct relationship that the smaller the precipitate, the smaller the grain size, Fig. 13.

The graph in Fig. 13 shows that for additions of inoculant up to 0.05 wt% Nb the reduction of the β-Fe precipitate is directly proportional to the refinement of the primary α-Al grain. However, when the addition is greater than 0.1%, the values remained practically equal to the previous level (already justified by the solubility of Nb in Al due to its melting temperature). Therefore, for addition values above 0.1 wt% Nb, this result should not be considered.

**Figure 14:**
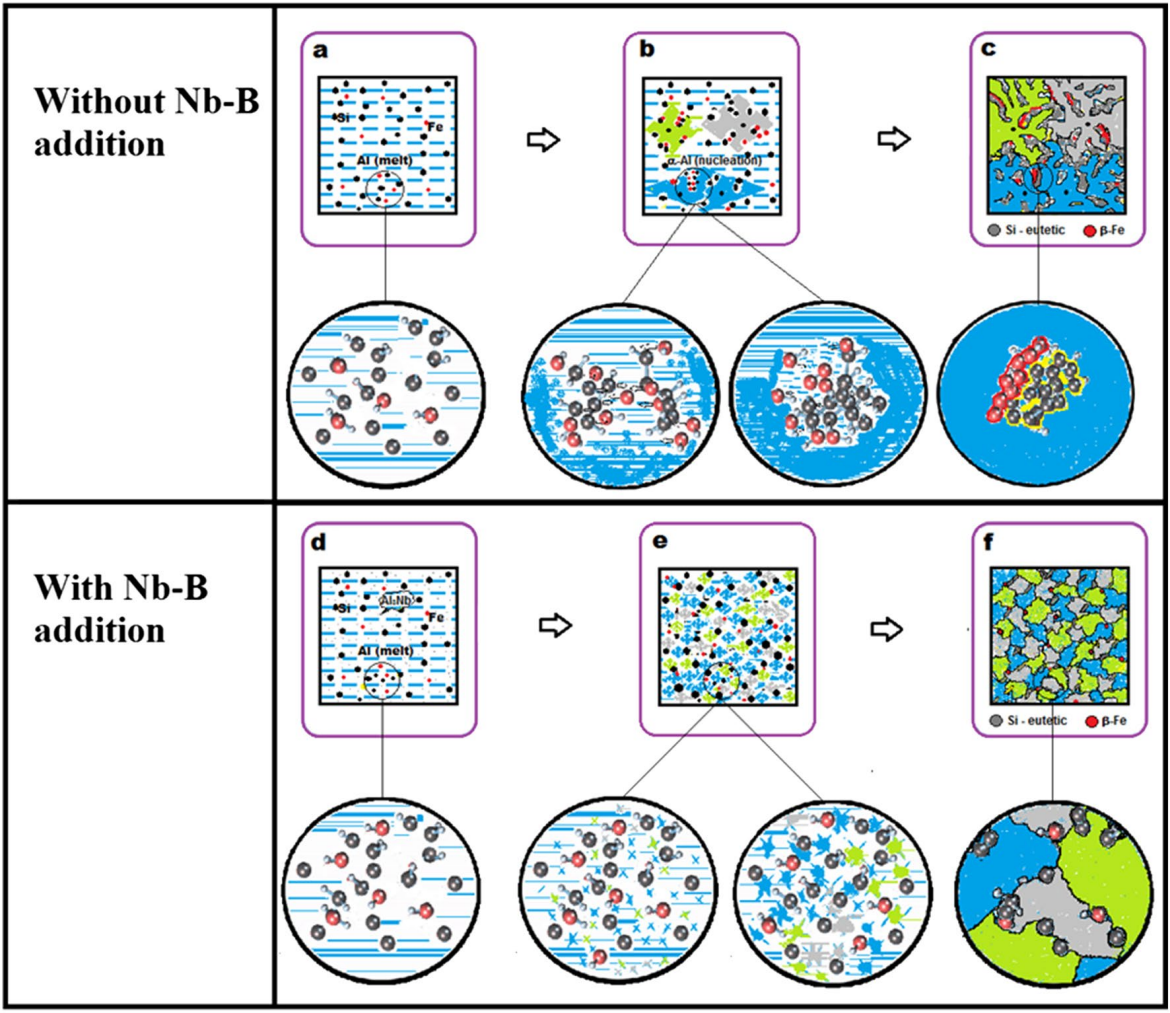
Illustration of the dispersion mechanism of β-Fe particles in the eutectic front of solidification of the material, without the addition of Nb + B (**a, b,** and **c**), with the addition of Nb + B (**d, e,** and **f**). Liquid metal (**a**, and **d**). Beginning of the solidification process and growth of the grain (**b**, and **e**). End of solidification (**c**, and **f**) (Microsoft Paint.Ink 2010).

With the information obtained in the study, it was possible to build a comparative schematic representation suggesting how the phenomenon of transformations (β-Fe) occurs without and with the addition of the Nb + B inoculant, as shown in Fig. 14.

Figure 14 shows the structural evolution in each solidification phase without and with the addition of the Nb + B inoculant. The addition caused an exponential increase in the nucleation points through the substrates Al_3_Nb and NbB_2_ (heterogeneous nucleation), forming a large number of simultaneous grains that ended up restricting the mobility of the solute elements, thereby decreasing their interconnectivity and the formation of agglomerates. Finally, the grains display a refined structure with reduced and spheroidized intermetallic precipitates, suggesting an improvement in the material's mechanical properties.

## Conclusions

This paper analysed the effect of 0 to 1 wt% Nb concentration on the morphology and size of the β-Fe precipitates in the Al-(7, 9 and 12) wt% Si-1wt.% Fe alloys. The addition of the Nb + B inoculant promoted the formation of Al_3_Nb and NbB_2_ substrates, which refined the size of the primary α-Al grain though the heterogeneous nucleation mechanism and modified the size and morphology of the β-Fe precipitates. Further investigations revealed that:

The addition of Nb + B is a powerful tool to improve the morphology and size of the β-Fe precipitates.

A slight addition of 0.02 wt% Nb and 0.0025 wt% B was enough to drastically change the β-Fe particles, which became very refined and spheroidized, in contrast to the rough morphology in the form of highly-faceted platelets, found in alloys without the addition of Nb + B.

The size and morphology of β-Fe precipitates in general follow an exponential decay curve with the increasing addition of Nb + B (Eq. 3).

The similarity between the primary α-Al grain's refinement curve and the change curve of the β-Fe precipitates’ morphology show a direct relationship between the events.

The melting temperature of the material turns out to be the limiting factor for the quantity in weight of the addition of the Nb + B inoculant. It is an important parameter for the industry in preparing the alloy during the material's melting.

The formation of the β-Fe precipitates generally follows an exponential decay with the addition of the Nb + B inoculant, being limited by the melting temperature of Al and it is directly proportional to the refinement of the primary α-Al grain.

The mechanism by which the phenomenon of the morphological transformation of the β-Fe precipitates occurs is due to the simultaneous formation of the several small dendritic arms that end up preventing the approach or grouping of the Fe solutes scattered in the material, getting trapped between the primary α-Al grains, thus inhibiting the formation of the coarse morphology in the form of highly-faceted platelets and turning into smaller and more dispersed precipitates in the structure of the material, with a spheroidized appearance.

The reduction in the size and morphology of the β-Fe precipitates suggests an improvement in the mechanical properties of the material.

The result of the study is a strong indication that the research involving alloys from recycling (Fe-critical) should continue, seeking out a reinforcement in their mechanical properties through the addition of Nb + B, as well as enabling their use in applications where strength and elasticity are required in the same product. Finally, this will contribute to improving the environment and reducing the cost of the final product due to the substitution of alloys from primary aluminium (with a higher cost)^1^.
